# Factors associated with hospital admission in patients reaching the emergency department with COPD exacerbation

**DOI:** 10.1186/2049-6958-7-6

**Published:** 2012-06-19

**Authors:** Maria Teresa García-Sanz, Carlos Pol-Balado, Concepción Abellás, Juan Carlos Cánive-Gómez, Diana Antón-Sanmartin, Francisco J González-Barcala

**Affiliations:** 1 Emergency Department, Salnés County Hospital, Ande-Rubiáns s/n, Vilagarcía de Arousa, Pontevedra, Spain; 2 Family and Community Medicine, Hospital Complex of Pontevedra, Pontevedra, Spain; 3 Pneumology Service, Hospital Complex of Pontevedra, Pontevedra, Spain

**Keywords:** Admission, COPD, Emergency Department, Exacerbation

## Abstract

**Background:**

The aim of this study was to determine the frequency of COPD exacerbations in our Emergency Department, as well as the hospitalization-related factors.

**Methods:**

Prospective observational study conducted in the Emergency Department of Salnés County Hospital among patients admitted for COPD exacerbation. Admission predictors were determined by multivariate analysis.

**Results:**

There were 409 exacerbations in 239 patients (79% male, mean age 75). 57% of exacerbations required hospitalization. Hospitalization-related factors were impaired oxygenation (*p* < 0.001), presence of neutrophilia (*p* < 0.01) and prescription of antibiotics in the Emergency Department (*p* < 0.05).

**Conclusions:**

COPD exacerbation accounts for over 1% of all visits to our Emergency Department. 57% of them required hospitalization. Impaired oxygenation, greater neutrophilia and prescription of antibiotics in the Emergency Department were associated with greater probability of admission.

## Background

Chronic Obstructive Pulmonary Disease (COPD) is a major cause of morbidity and mortality worldwide [1]. It presents exacerbations that often lead the patient to the Emergency Department (ED) and to hospitalization, contributing significantly to higher health care costs [2]. In Spain, COPD prevalence in adults aged 40-69 is 9.1% [3]. The disease ranks fourth among causes of mortality [4] and it is responsible for 7% of hospital admissions [5]. COPD-related costs in 1998 were estimated at about € 1,210 million [6].

Despite the large economic impact posed by COPD exacerbations, there is a still ongoing debate about the predictors of higher likelihood of hospitalization in patients with COPD exacerbation which have received hospital care in an ED [7-9].

Latest studies have shown a significant increase in hospital admissions for chronic obstructive respiratory disease in recent years in our region [10].

The aim of this study was to determine the frequency of COPD exacerbations in our ED, as well as the hospitalization-related factors.

## Methods

This is a prospective observational study conducted in the ED of Salnés County Hospital, a 100-bed facility providing health coverage to 80,000 people , for one year (from March 1, 2009 to February 28, 2010), among patients admitted for COPD exacerbations. Included in the study there were patients with a COPD diagnosis confirmed by a doctor, and those patients with consistent clinical, radiological and epidemiological manifestations , typical symptoms, and smoking history greater than 10 pack years. The information was obtained from medical records and by interviewing patients during their stay in the ED. COPD severity was determined on the basis of the GOLD guidelines [11]. COPD exacerbation was defined as the sustained worsening of the patient’s condition, from the stable state and beyond the normal day-to-day variations, that is acute in onset and necessitates a change in regular medication in a patient with underlying COPD [12]. Hospital admission was defined as admission to inpatient unit, observation unit, or intensive care unit after receiving the first care in the ED [8].

The following variables were analyzed:

1. Patient characteristics: age, gender, years of COPD evolution, that is the time elapsed from the COPD diagnosis or from the start of compatible symptoms referred by the patient till the moment of inclusion in the study; smoking status, categorized as “never smoker”, “active smoker”, “passive smoker” and “former smoker”; comorbidities; employment status, employed, unemployed, retired; reason for retirement, age, COPD, other diseases, and social support.

2. Characteristics of the underlying disease: COPD severity [11], patient regular treatment, previous treatment with systemic steroids, oxygen therapy at home, ED care and hospital admissions in the year before and in the year when the patient was enrolled in the study.

3. Data of current exacerbation: O_2_ saturation measured by pulse oximetry and vital signs, considering systolic hypotension SBP < 100 mm Hg, diastolic hypotension DBP < 60 mm Hg and tachycardia heart rate > 100 [13,14]. Baseline oxygenation was stratified into two categories based on SaO_2_/FiO_2_ ratio, setting the cut off value at 90% saturation breathing room air, which corresponds to a value of 428.6. The analytical parameters included (hemoglobin, leukocytosis, neutrophilia and urea) were stratified into three categories: normal, high, low , according to the reference values in our medical laboratory. With regard to severity of the episode, the priority of care was based on the MTS (Manchester Triage System), which classifies the patient into five categories, enabling care prioritization according to patient severity: 1 = immediate: immediate attention; 2 = very urgent: care delayed up to 10 minutes; 3 = urgent: 60 minutes; 4 = standard: 120 minutes; 5 = non-urgent: 240 minutes [15].

4. Treatments provided in the ED, and finally.

5. Data on the evolution of the exacerbation: length of stay in ED, patient destination, discharge or admission criteria.

### Statistical analysis

The variables included in the study were analyzed on the basis of hospital admission for COPD exacerbation. Fisher’s exact test and Chi-square test were used to compare categorical variables. T-Student test was used to analyze continuous variables following normal distribution, as well as Levene test to verify the homogeneity of the variances. Mann-Whitney U-test was used for non-Gaussian distributions.

Likelihood of hospitalization was estimated by multivariate logistic regression, including in the analysis those variables that showed differences between patients admitted and not admitted with *p* < 0.1. All data were analyzed with SPSS 15 for Windows.

## Results and discussion

In the year of study, 409 exacerbations were registered in 239 patients (representing 1.07% of all emergencies treated), with a rate of 1.12 episodes-day. 69% of patients came only once to the hospital. 79% out of 239 patients were male. The mean age was 75 years (ranging 41-96, SD 9.51). 57% out of all exacerbations required admission. 11% out of all admissions were done in home care regime. Only one case required admission to the Intensive Care Unit (ICU). The admission decision was based on exacerbation severity as per clinical and analytical criteria in 61 patients, and the presence of decompensated comorbidity in 81 cases. The most frequent discharge reasons were mild COPD exacerbation or absence of respiratory failure after arterial blood gas measurement (data not shown). Univariate analysis revealed a higher frequency of hospital admissions in the following cases: those of highest priority for initial care according to the Manchester Scale (Types 1 and 2 account for most admissions); those with impaired oxygenation, higher respiratory effort, or requiring O_2_ administration; those receiving bronchodilator or steroid treatment in the ED; and those with respiratory-infection related COPD exacerbation, with longer ED stays, or with a history of COPD-exacerbation related admissions (Table 1). Hospitalization-related factors in multivariate analysis were impaired oxygenation, presence of neutrophilia and administration of antibiotics in the ED (Table 2).

**Table 1:** Demographic and clinical characteristics of patients assessed by the ED for COPD exacerbation, sorted by hospital admission

**Predictors**	**N**	**Not admitted**	**Admitted**	p
Gender				
Male	188 (78.7%)	78 (41.5%)	110 (58.5%)	NS
Female	51 (21.3%)	22 (43.1%)	29 (56.9%)	
Age				
< 65 years	37 (15.5%)	16 (43.2%)	21 (56.8%)	NS
65-75 years	74 (31.1%)	30 (40.5%)	44 (59.5%)	
>75 years	127 (53.4%)	54 (42.5%)	73 (57.5%)	
Manchester				
1 and 2	98 (41.17%)	24 (24.5%)	74 (75.5%)	***
3	100 (42.0%)	44 (43.6%)	56(56.4%)	
4 and 5	40 (16.8%)	32 (80.0%)	8 (20.0%)	
Gold				
0	102 (42.7%)	52 (51.0%)	50 (49.0%)	*
1 and 2	61 (25.5%)	26 (42.6%)	35 (57.4%)	
3 and 4	76 (31.8%)	22 (28.9%)	54 (71.1%)	
Smoker				
No	50 (23.0%)	24 (48.0%)	26 (52.0%)	NS
Yes	34 (15.7%)	16 (47.1%)	18 (52.9%)	
Former smoker	130 (59.9%)	51 (39.2%)	79 (60.8%)	
Passive smoker	3 (1.4%)	1 (33.3%)	2 (66.7%)	
Tachypnea				
No	149 (62.3%)	76 (51.0%)	73 (49.0%)	***
Yes	90 (37.7%)	24 (26.7%)	66 (73.3%)	
SBP				
< 100	5 (2.1%)	2 (40.0%)	3 (60.0%)	NS
100-140	110 (46.2%)	47 (42.7%)	63 (57.3%)	
>140	123 (51.7%)	51 (41.5%)	72 (58.5%)	
DBP				
<60	31 (13.5%)	11 (35.5%)	20 (64.5%)	NS
60-90	174 (76.0%)	77 (44.3%)	97 (55.4%)	
>90	24(10.5%)	12 (35.3%)	22 (64.7%)	
T^a^				
<37°C	172 (72.0%)	79 (45.9%)	93 (54.1%)	*
>37C	67 (28.0%)	21 (31.3%)	46 (68.7%)	
HR				
<100	156 (65.5%)	74 (47.4%)	82 (56.2%)	*
>100	82 (34.5%)	25 (30.5%)	57 (69.5%)	
SaO_2_/FiO_2_				
<428.6	84 (35.1%)	8 (9.5%)	76 (90.5%)	***
>428.6	155 (64.9%)	92(59.4%)	63 (40.6%)	
O_2_ at Home				
No	196 (82.0%)	85 (43.4%)	111 (56.6%)	NS
Yes	43 (18.0%)	15 (34.9%)	28 (65.1%)	
Baseline Treatment				
Oral Corticosteroids	16 (6.7%)	6 (37.5%)	10 (62.5%)	NS
Anticholinergics and/or saba and/or laba	26 (11.3%)	8 (30.8%)	18 (69.2%)	
Anticholinergics and/or saba and/or laba + Inhaled Corticosteroids	140 (58.3%)	63 (45.0%)	77 (55.0%)	
None of these (ALT, Theophylline..)	55 (22.9%)	23 (41.8%)	32 (58.2%)	
ED Treatment				
Antibiotics				
No	102 (43.4%)	61 (59.8%)	41 (40.2%)	***
Yes	133 (56.6%)	39 (29.3%)	94 (70.7%)	
O_2_				
No	26 (11.1%)	23 (88.5%)	3 (11.5%)	***
Yes	209 (88.9%)	77 (36.8%)	132 (63.2%)	
Systemic Corticosteroids				
No	64 (27.2%)	40 (62.5%)	24 (37.5%)	***
Yes	171 (72.8%)	60 (35.1%)	111 (64.9%)	
Inhaled/Nebulized Cortocosteroids				
No	183 (76.6%)	85 (46.4%)	98 (53.6%)	**
Yes	56 (23.4%)	15(26.8%)	41 (73.2%)	
Any Nebulized Drugs				
No	44 (18.4%)	22 (50.0%)	22 (50.0%)	NS
Yes	195 (81.6%)	78 (40%)	117 (60%)	
Beta-agonists				
No	39 (16.3%)	24 (61.5%)	15 (38.5%)	**
Yes	200 (83.7%)	76 (38.0%)	124 (62.0%)	
Anticholinergics				
No	40 (17.1%)	23 (57.5%)	17 (42.5%)	*
Yes	194 (82.9%)	77 (39.7%)	117 (60.3%)	
Anti-Leukotrienes				
No	232 (98.7%)	100( 43.1%)	132 (56.9%)	NS
Yes	3 (1.3%)	0 (0.0%)	3 (100%)	
Theophyllines				
No	231 (98.3%)	99 (42.9%)	132 (57.1%)	NS
Yes	4 (1.7%)	1 (25.0%)	3 (75.0%)	
Mucolytics				
No	206 (87.7%)	87 (42.2%)	119 (57.8%)	NS
Yes	29(12.3%)	13 (44.8%)	16 (55.2%)	
Month				
January-March	78 (32.6%)	33 (42.3%)	45 (57.7%)	NS
April-June	59 (24.7%)	23 (39.0%)	36 (61.0%)	
July-September	52 (21.8%)	23 (44.2%)	29 (55.8%)	
October-December	50 (20.9%)	21 (42.0%)	29 (58.0%)	
Day of the Week				
Monday-Thursday	150 (62.8%)	62 (41.3%)	88 (58.7%)	NS
Friday-Sunday	89 (37.2%)	38 (42.7%)	51 (57.3%)	
Time				
0-8:00	38 (15.9%)	12 (31.6%)	26 (68.4%)	NS
8:00-16:00	113 (47.3%)	48 (42.5%)	65 (57.5%)	
16:00-24:00	88 (36.8%)	40 (45.5%)	48 (54.5%)	
Hospital Readmissions				
None	201 (84.1%)	90 (44.8%)	111(55.2%)	*
Any (Early or Late)	38 (15.9%)	10 (26.3%)	28 (73.7%)	
ED Readmissions				
None	228 (95.4%)	96 (42.1%)	132 (57.9%)	NS
Any (Early or Late)	11 (4.6%)	4 (36.4%)	7 (63.6%)	
Number of Admissions				
None	82 (34.3%)	79 (96.3%)	3 (3.7%)	***
One	120 (50.2%)	12 (10.0%)	108 (90.0%)	
Two or More	37 (15.5%)	9 (24.3%)	28 (75.7%)	
Number of Emergencies				
One	166 (69.5%)	64 (38.6%)	102 (61.4%)	NS
Two	33 (13.8%)	16 (48.5%)	17 (51.5%)	
Three or More	40 (16.7%)	20 (50.0%)	20 (50.0%)	
Length of the Crisis (Hours)				
< 12	23 (10.8%)	9 (39.1%)	14 (60.9%)	NS
12-24	35 (16.4%)	10 (28.6%)	25 (71.4%)	
24-72	29 (13.6%)	11 (37.9%)	18 (62.1%)	
>72	126 (59.2%)	58 (46.0%)	68 (54.0%)	
Length of ED Stay (Hours)				
< 4	63 (27.8%)	38 (60.3%)	25 (39.7%)	***
4-8	50 (22.0%)	23 (46.0%)	27 (54.0%)	
8-18	58 (25.5%)	20 (34.5%)	38 (65.5%)	
>72	126 (24.7%)	11 (19.6%)	45 (80.4%)	
Hemoglobin				
Low	106 (44.7%)	46 (43.4%)	60 (56.6%)	NS
Normal	128 (54%)	52 (40.6%)	76 (59.4%)	
High	3 (1.3%)	0 (0.0%)	3 (100.0%)	
Leukocytes				
Leukopenia	5 (2.1%)	3 (60.0%)	2 (40.0%)	***
Normal	154 (64.4%)	79 (51.3%)	75 (48.7%)	
Leukocytosis	80 (33.5%)	18 (22.5%)	62 (77.5%)	
Neutrophil count				
Low	3 (1.3%)	1 (33.3%)	2 (66.7%)	***
Normal	127 (53.1%)	69 (54.3%)	58 (45.7%)	
High	109 (45.6%)	30 (27.5%)	79 (72.5%)	
Urea				
Low	4 (1.8%)	2 (50.0%)	2 (50.0%)	NS
Normal	120 (52.6%)	54 (45%)	66(55.0%)	
High	104 (45.6%)	33 (31.7%)	71 (68.3%)	
Emergencies the Year Before				
None	150 (62.7%)	59 (39.3%)	91 (60.7%)	NS
One	37 (15.5%)	19 (51.4%)	18 (48.6%)	
Two or More	52 (21.8%)	22 (42.3%)	30 (57.7%)	
Hospital Admissions the Year Before				
None	178 (75.1%)	75 (42.1%)	103 (57.9%)	NS
One	37 (15.6%)	16 (43.2%)	21 (56.8%)	
Two or More	22 (9.3%)	7 (31.8%)	15 (68.2%)	

*p < 0.05.

**p < 0.01.

*** *p* < 0.001. NS = not significant.

**Table 2:** Predictors of hospital admission according to multivariate analysis

**Factors**	**OR**	**CI 95%**	**p**
Neutrophilia	3.15	(1.570, 6.335)	**
SaO_2_/ FiO_2_ > 428.6	0.080	(0.034, 0.187)	***
Antibiotics	2.175	(1.081, 4.376)	*

*p < 0.05.

**p < 0.01.

*** *p* < 0.001.

Exacerbations are a fundamental aspect of COPD-generated costs [16]. The decision to admit a patient with COPD exacerbation is based on the interpretation of a series of clinical data, such as severity of dyspnea, respiratory failure, poor response to treatment during the stay in the ED, and presence of pneumonia or other comorbidities [17]. In our patients, as in other studies, the decompensation of underlying diseases is a frequent cause of hospitalization [18]. Determinants of hospital admission in COPD exacerbations in our environment depend mainly on some characteristics of the exacerbation, such as impaired oxygenation and the presence of neutrophilia. The other factor which proved to be an independent predictor of hospital admission was the decision to administer antibiotics by the ED, which also depends - at least in part - on the exacerbation severity determined by the MD in charge.

Respiratory failure is one of the criteria that lead to the decision to grant hospital admission, together with exacerbation severity and even the need for mechanical ventilation in patients with COPD exacerbations [19].

In our study, oxygenation - assessed by the SaO_2_/FiO_2_ ratio - was lower in patients who were admitted, indicating the severity of the exacerbation or of the underlying disease [19].

Given its simplicity, pulse oximetry seems to be a particularly useful method to assess oxygenation in patients with COPD exacerbation: it does not require special training, is reliable in the range of 80-100% saturation and, although it is not a substitute for blood gas measurement, it provides real time, continuous and non-invasive monitoring of heart rate, can alert on tissue perfusion decreases [20], and detect hypoxia with high sensitivity and specificity [21].

Although pulse oximetry provides guidance on the initial need for oxygen therapy, arterial blood gas measurement is essential to assess potential hypercapnia and acidosis [11].

Inflammatory markers could also be used as a parameter to assess the severity of COPD exacerbations [22]. One of the systemic markers of inflammation is the neutrophil count in peripheral blood, which is higher in bacterial exacerbations [23]. Some authors report significant relationship between neutrophilia in peripheral blood, exacerbation severity and hospital admission frequency in COPD exacerbations [24,25]. In our case, neutrophil count in peripheral blood was significantly higher among those admitted, so neutrophilia could be a particularly important indicator in the initial exacerbation assessment in those patients.

The causes of exacerbations are not always known and, in connection with the latter, also the optimal treatment, that should logically be different according to exacerbation etiology. In our study, the initial decision by the ED doctor to start an antibiotic treatment appears to be associated with a higher likelihood of hospital admission, regardless of both patient baseline and exacerbation severity. Since the indication of antibiotic treatment in COPD exacerbations is not clearly established [26-28], this decision could be based on clinical criteria [29], and even in those cases where the likelihood of bacterial infectious etiology is not evident, some doctors may consider antibiotic prescription to be safer [28].

Antibiotic treatment seems to be beneficial in most severe exacerbations and in those cases with high clinical likelihood of infection [30-32]. Also, antibiotic prescription in COPD exacerbations which require hospital admission is widespread [26]. These facts may condition the prescription of antibiotic treatment in the ED to change the perception of COPD exacerbation severity by the doctors who eventually request patient admission, regardless of objective criteria: the start of antibiotic treatment in the ED means to increase the likelihood of hospital admission by more than twice.

## Conclusions

In conclusion, COPD exacerbations account for over 1% of all visits to our ED, 57% of which will require hospitalization. Impaired oxygenation, higher peripheral blood neutrophilia and antibiotic prescription in the ED are associated with a greater likelihood of hospitalization in COPD exacerbations assessed in the ED.

## Competing interests

The authors declare that they have no competing interests.
